# Supplementary material for: Antimicrobial Peptides Epinecidin-1 and Beta-Defesin-3 Are Effective against a Broad Spectrum of Antibiotic-Resistant Bacterial Isolates and Increase Survival Rate in Experimental Sepsis
Source: Antibiotics (Basel). 2022 Jan 9;11(1):76. doi: 10.3390/antibiotics11010076 (PMC8773371; doi:10.3390/antibiotics11010076)

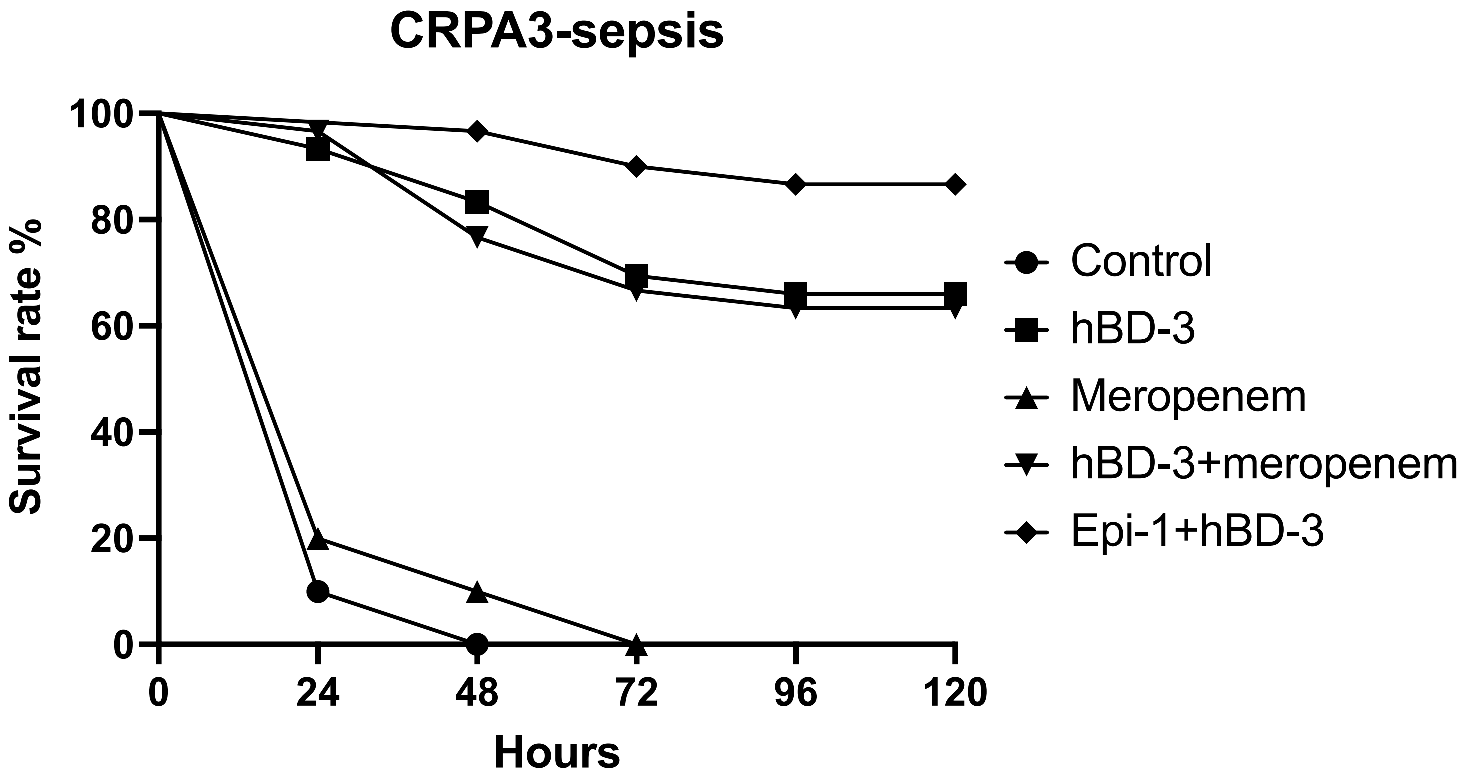
CRPA3-sepsis
Survival rate %
100
80
60
40
20
0
0
24
48
72
96
120
Hours
Control
hBD-3
Meropenem
hBD-3+meropenem
Epi-1+hBD-3

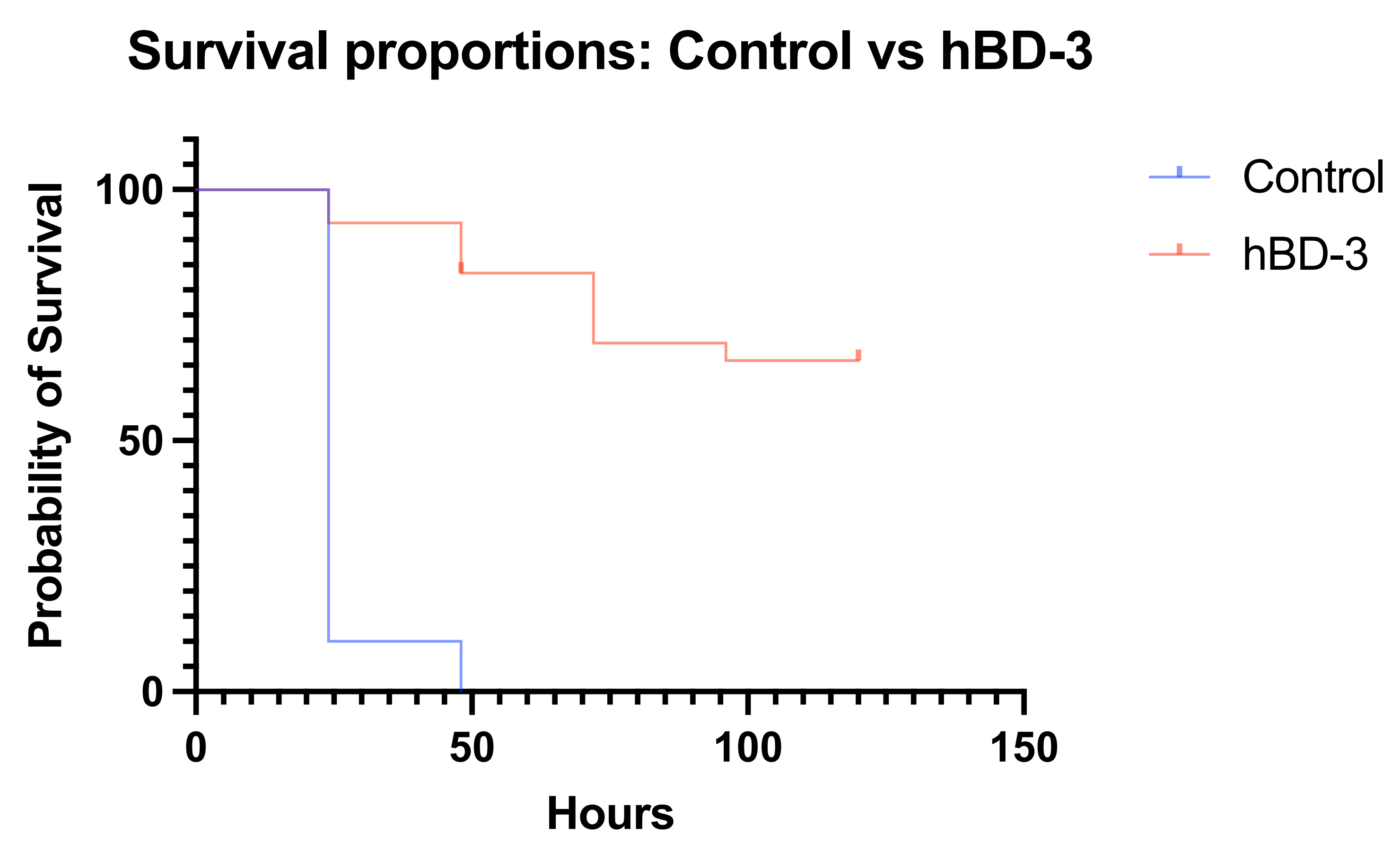
Survival proportions: Control vs hBD-3
Probability of Survival
100
50
0
0
50
100
150
Hours
Control
hBD-3

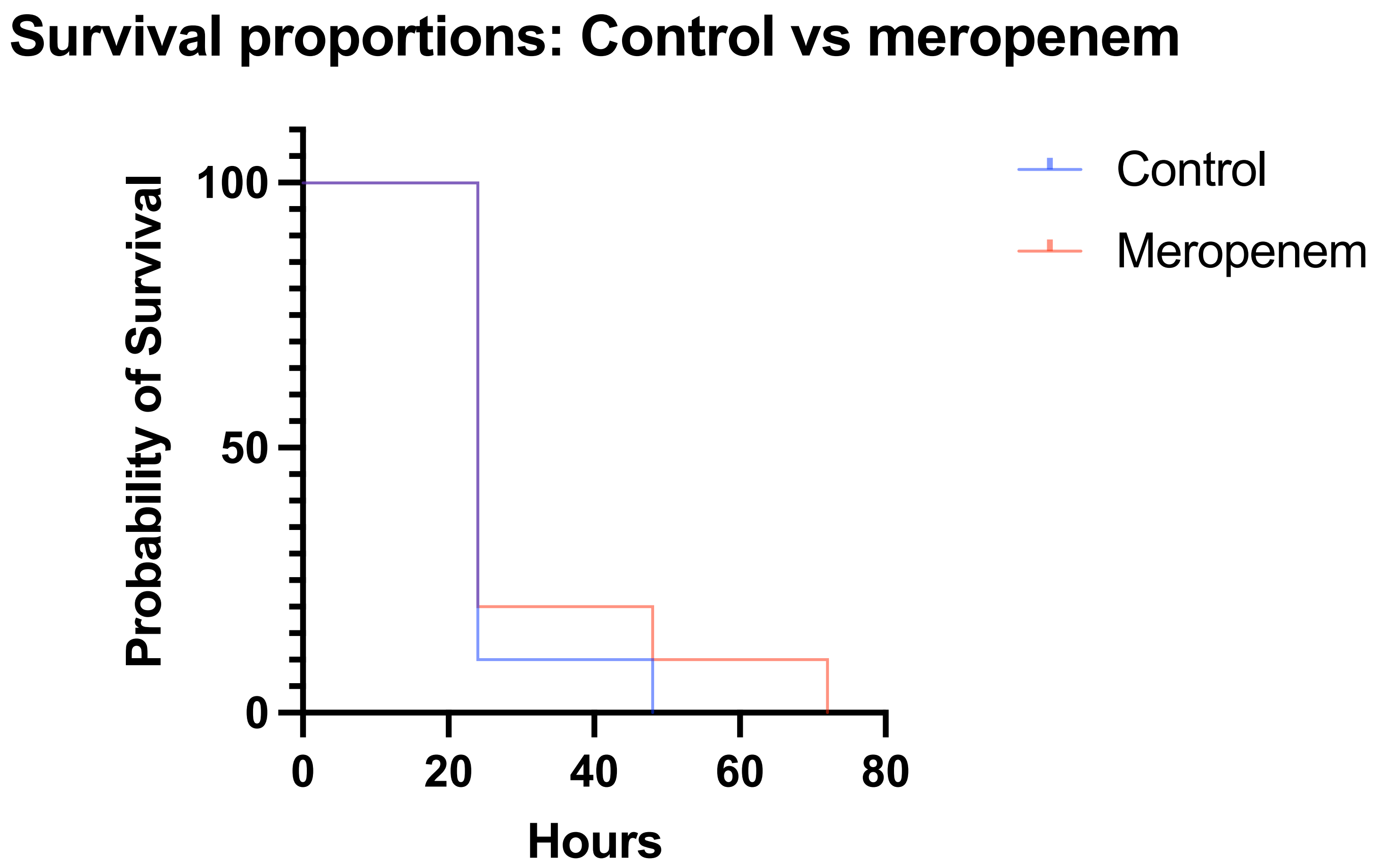
Survival proportions: Control vs meropenem
Probability of Survival
100
50
0
0
20
40
60
80
Hours
Control
Meropenem

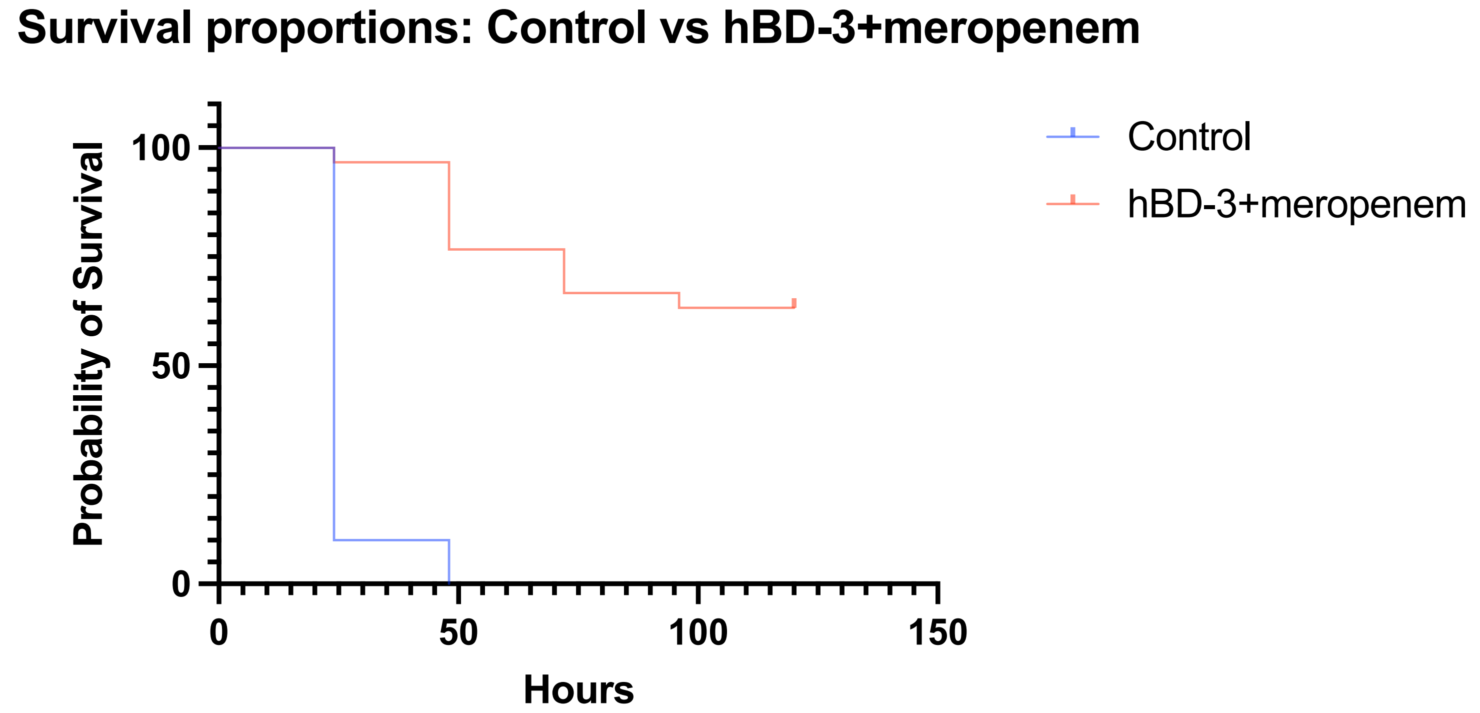
Survival proportions: Control vs hBD-3+meropenem
Probability of Survival
100
50
0
0
50
100
150
Hours
Control
hBD-3+meropenem

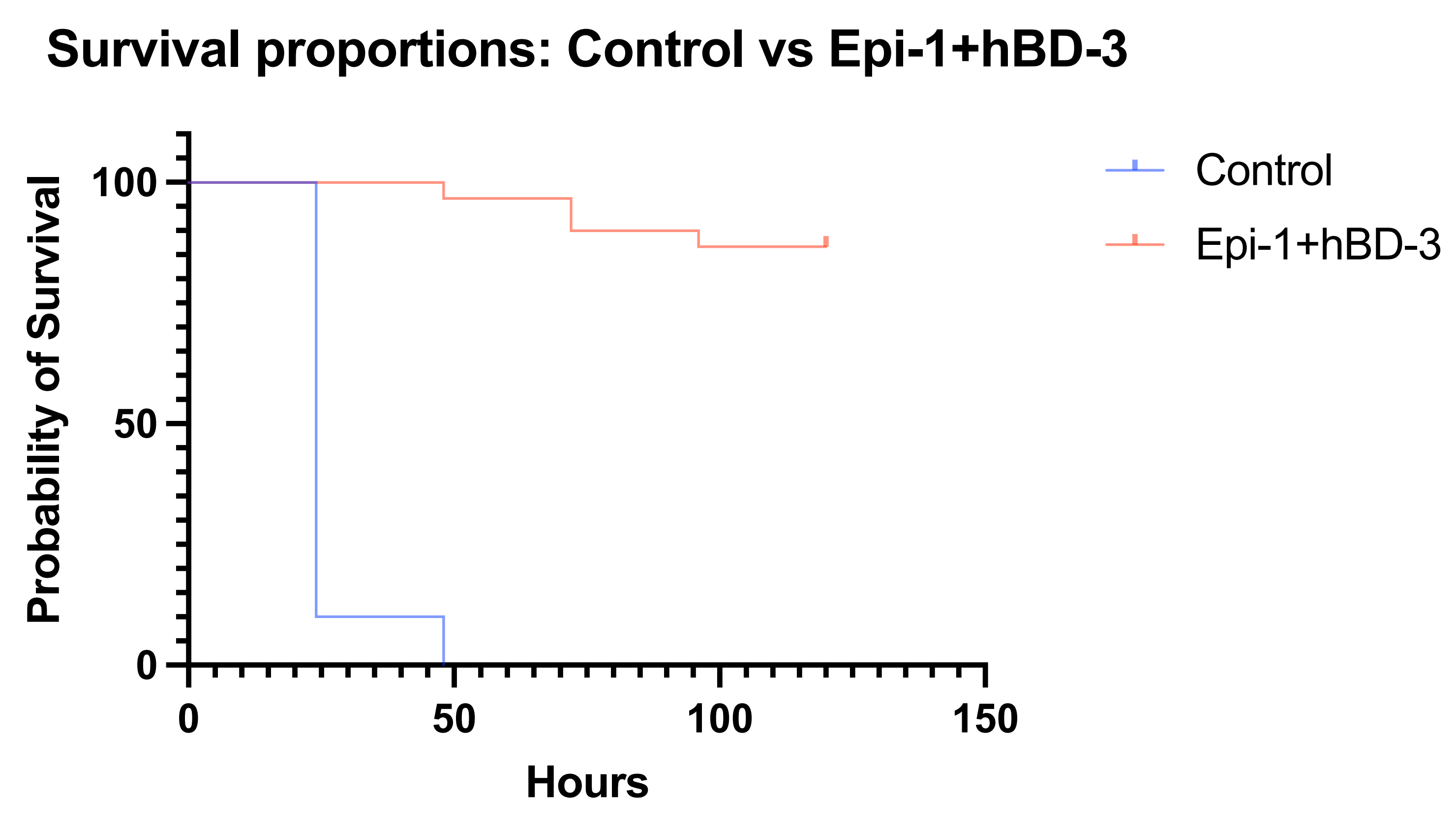

Survival proportions: Control vs Epi-1+hBD-3
Probability of Survival
100
50
0
0
50
100
150
Hours
Control
Epi-1+hBD-3

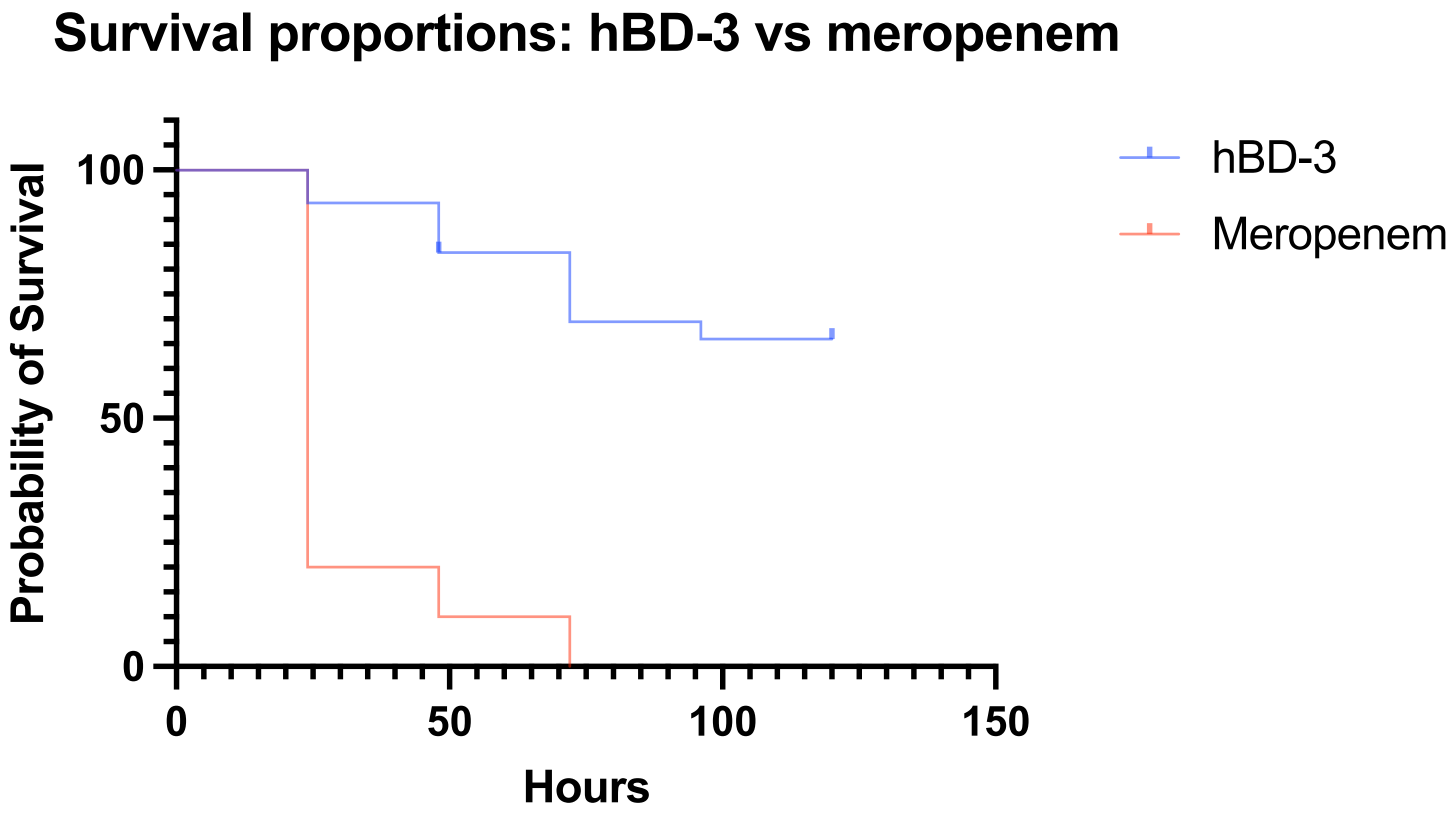
Survival proportions: hBD-3 vs meropenem
Probability of Survival
100
50
0
0
50
100
150
Hours
hBD-3
Meropenem

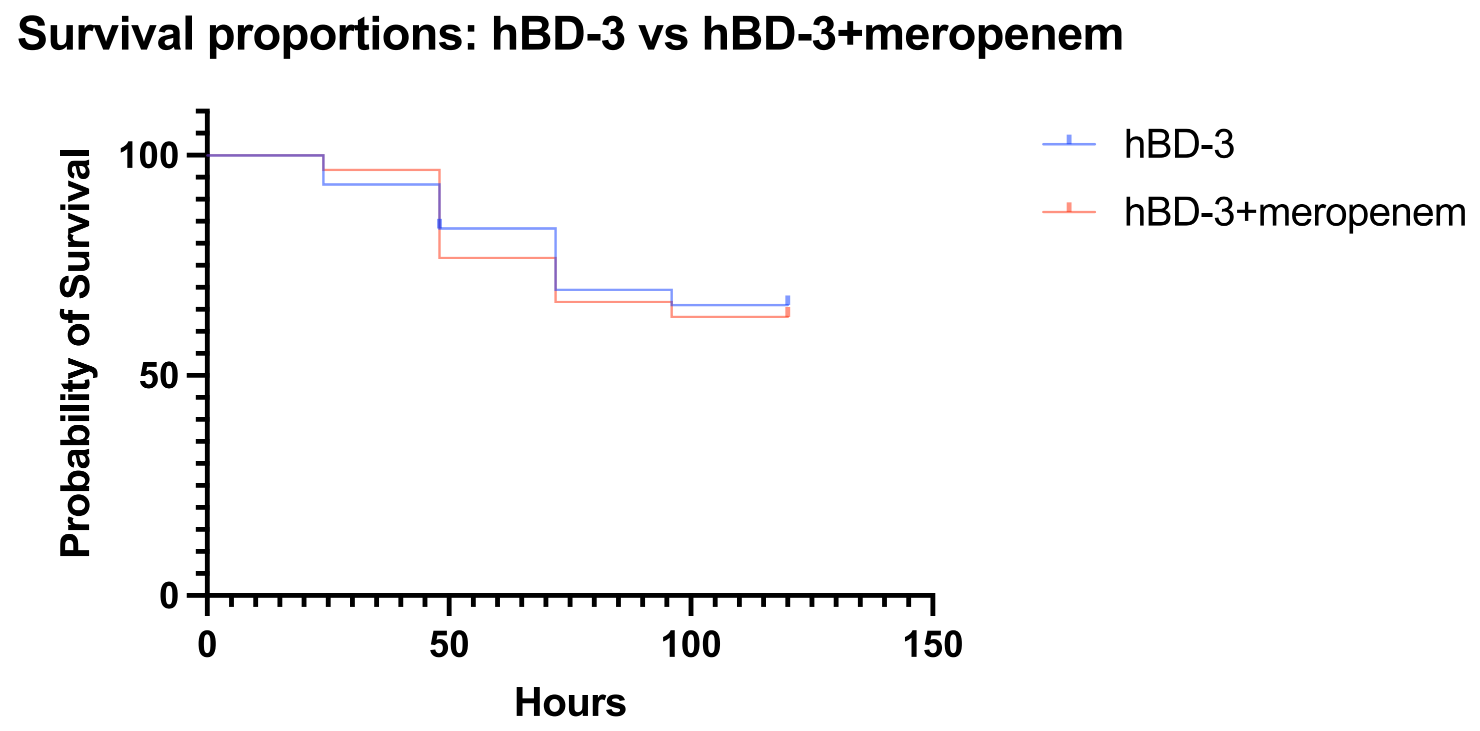
Survival proportions: hBD-3 vs hBD-3+meropenem
Probability of Survival
100
50
0
0
50
100
150
Hours
hBD-3
hBD-3+meropenem

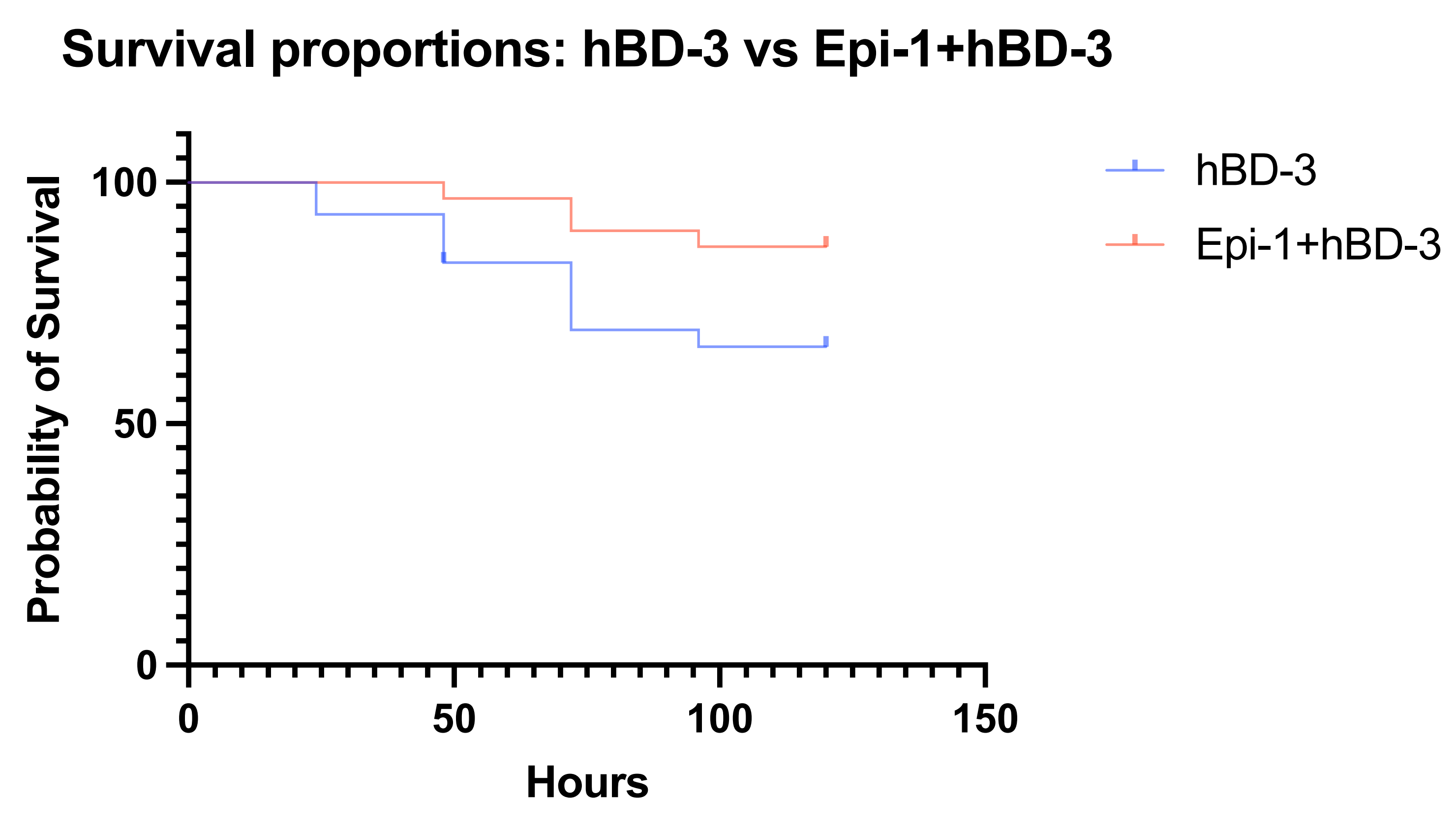

Survival proportions: hBD-3 vs Epi-1+hBD-3
Probability of Survival
100
50
0
0
50
100
150
Hours
hBD-3
Epi-1+hBD-3

Survival proportions: hBD-3+meropenem vs Epi-1+hBD-3

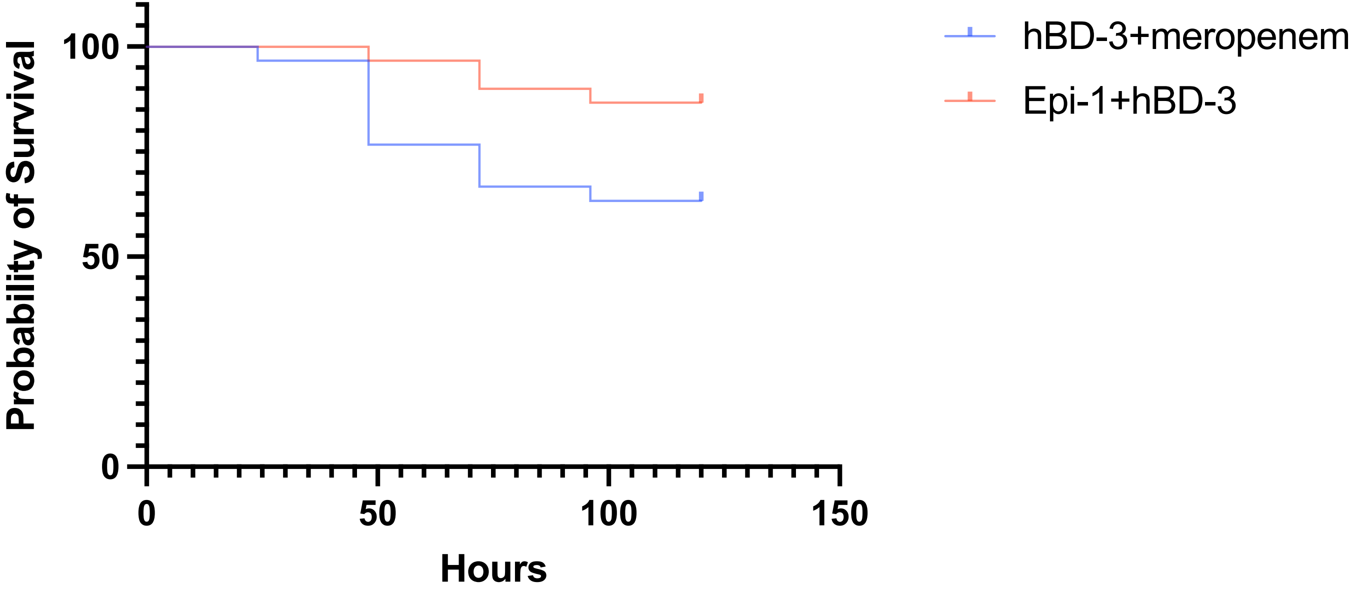

Supplement: Supplementary file 1 [file antibiotics-11-00076-s001.zip › CRPA3 in vivo/CRPA3 graphs.pdf]
